# A Topic Clustering Approach to Finding Similar Questions from Large Question and Answer Archives

**DOI:** 10.1371/journal.pone.0071511

**Published:** 2014-03-04

**Authors:** Wei-Nan Zhang, Ting Liu, Yang Yang, Liujuan Cao, Yu Zhang, Rongrong Ji

**Affiliations:** 1 Research Center for Social Computing and Information Retrieval, Harbin Institute of Technology, Harbin, Heilongjiang, China; 2 School of Computing, National University of Singapore, Singapore, Singapore; 3 School of Information Science and Technology, Xiamen University, Xiamen City, Fujian, China; 4 Department of Cognitive Science, Xiamen University, Xiamen City, Fujian, China; University of Adelaide, Australia

## Abstract

With the blooming of Web 2.0, Community Question Answering (CQA) services such as Yahoo! Answers (http://answers.yahoo.com), WikiAnswer (http://wiki.answers.com), and Baidu Zhidao (http://zhidao.baidu.com), etc., have emerged as alternatives for knowledge and information acquisition. Over time, a large number of question and answer (Q&A) pairs with high quality devoted by human intelligence have been accumulated as a comprehensive knowledge base. Unlike the search engines, which return long lists of results, searching in the CQA services can obtain the correct answers to the question queries by automatically finding similar questions that have already been answered by other users. Hence, it greatly improves the efficiency of the online information retrieval. However, given a question query, finding the similar and well-answered questions is a non-trivial task. The main challenge is the word mismatch between question query (*query*) and candidate question for retrieval (*question*). To investigate this problem, in this study, we capture the word semantic similarity between *query* and *question* by introducing the topic modeling approach. We then propose an unsupervised machine-learning approach to finding similar questions on CQA Q&A archives. The experimental results show that our proposed approach significantly outperforms the state-of-the-art methods.

## Introduction

With the proliferation and growth of Web 2.0, CQA services have become the integral part of information and knowledge acquisition. It provides a main platform for information seekers to post their specific questions in a wide range of topics and obtain answers, comments and other interactions, such as voting and rating, provided by other users. CQA services provide a real space for online communications. Either the topics or the questions and answers are posted by human, which we called user generated content (UGC). Hence the quality of Q&A pairs in CQA repositories is high. Over time, a large amount of Q&A pairs with high quality devoted by human intelligence have been accumulated as comprehensive knowledge base.

One of the most popular CQA services is Yahoo! Answers (http://answers.yahoo.com/), which is launched on December 13, 2005. It greatly facilitates the users to acquire knowledge and information [1]–[5] online. In Yahoo! Answers, each question has two parts, one is question title, and the other is question description. Table 1 shows an example of the Q&A pair in Yahoo! Answers.

**Table 1:** An example of the Q&A pair in Yahoo! Answers repository.

**Question Title:**
What phone is best iPhone 5 or Samsung galaxy s3?
**Question Content:**
What phone is best to have iPhone 5 or Samsung galaxy s3? I've heard that the Samsung galaxy battery only lasts for 6 hrs and what's the best for apps?
**Best Answer:**
Galaxy s3 hands down. Galaxy s3 features. Quad core processor. Several days of battery life. Flash. Better camera with more features such as burst mode. Will soon get Android 4.1 jellybean. Turn by turn voice navigation (said to be superior to apple maps) Built in FM radio. Micro SD card. Double your storage size. Multiple buttons. 4.8 inch super AMOLED plus display. More durable. Uses polycarbonate instead of glass…

For the special form of CQA questions (as shown in Table 1), the QA task is transferred from searching candidate documents and extracting answers to finding similar and well answered questions.

As the complexity of the CQA questions and the variety of the users' expressions, the traditional Bag-of-Word (BoW) methods [6]–[11] are failing to measure the semantic similarity between *query* and *questions*. Meanwhile, syntactic based question match approaches [12], [13] also have their limitations on capturing the semantic similarity of CQA questions.

To address the above problems, we propose a topic-based semantic similarity computing approach to finding similar questions in CQA archives. We first utilize the topic model [14] to capture question topic information and map the Q&A pairs into the topic space. We then propose an unsupervised machine learning approach to explore the similarity between the *query* and the *question* in topic space and automatically gather the similar questions from the Q&A repository.

The remainder of the paper is organized as follows. Section Related Work introduces the related work. Section Background on Latent Dirichlet Allocation gives a briefly introduction about the Latent Dirichlet Allocation (LDA). Section Topic Clustering Approach presents the topic-based clustering approach to finding similar questions. Section Experiments gives the experimental results and error analysis, followed by the conclusion and future work in Section Conclusions.

## Methods

### Related Work

Text similarity computing is widely applied in question answering (QA). In the TREC (Text Retrieval Conference, http://trec.nist.gov/) QA track, the QA systems need to capture the similarity between the questions and the candidate documents, and then return the relevant documents. In the application of the interactive QA, users input their questions in natural languages; the system then searches the candidate documents online and returns the answer list by computing the similarity between the users' questions and candidate answers.

Question similarity computing can be measured in three dimensions, e.g., lexical, syntactic and semantic.

The BoW method is a kind of lexical based method in similarity computing. It obtains the similarity between two questions by computing the number of the same words in them. The classical BoW methods include Jaccard similarity coefficient, inverse document frequency (IDF) overlap method [15] and phrase overlap method [16]. Moreover, the Vector Space Model (VSM) which is a typical BoW model is based on term frequency (TF) and inverse document frequency (IDF) [17], [18]. Despite their successes, the BoW methods only capture the string matching features in computing text similarity. Meanwhile, they also overlook the word sense [19], word order [20] and syntactic [21] information.

The syntactic-based methods focus on the similarity of syntactic structure. They consider the similarities of both the lexical and the syntactic structure. For example, they used tree kernel methods to calculate the common sub-trees between two questions [13], [19], [21], [22]. However, first, the syntactic tree matching approaches are so strict that the data sparse problem may occur. Second, it's hard to identify the similar substructure without fuzzy matching. However, even the fuzzy matching based approach [13] also cannot well capture the semantic and topic level similarity between two questions.

Furthermore, [23] and [24] compared the four different retrieval models, i.e., vector space model, okapi, language model and translation model for question retrieval in archived CQA data. The experimental results revealed that the translation model outperforms the other models. The reference [4] proposed a term weighting approach for question retrieval on CQA. Although the translation model can bridge the lexical gap between the *query* and *questions*, it can only capture the lexical level similarity.

The existing topic-based similar question finding approach [25] represented the *questions* by reformulating them to a topic and focus structure. They then utilized the MDL based tree cut model to select relevant words. At last, they used the similar topics to finding similar questions. However, as the CQA questions are more complex, it is hard to identify the question topics.

To investigate the above problem, in this study, we employ the Latent Dirichlet Allocation (LDA) model to identifying the question topics. We then propose a topic clustering based approach to finding similar questions, which can effectively measure the topic level semantic similarity between two questions.

### Background on Latent Dirichlet Allocation

**Figure 1:**
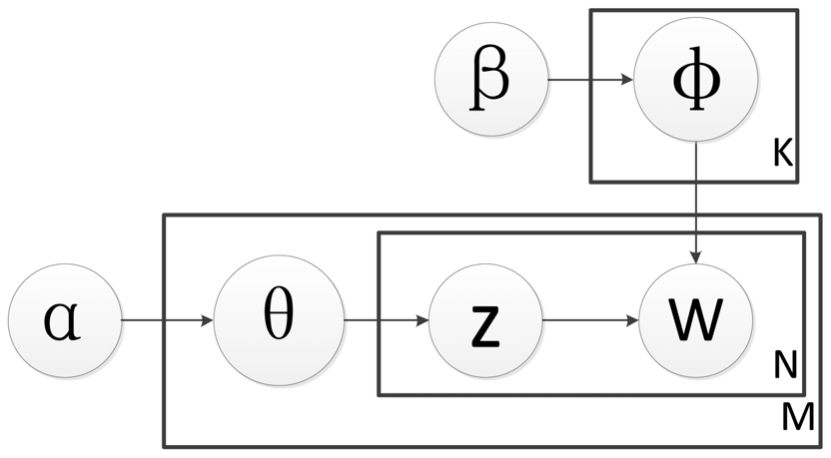
The graphical representation of the LDA model.

Latent Dirichlet Allocation (LDA) is first proposed by [14]. As a kind of probabilistic topic model, LDA is a generative model which can be used to inference the observation data with a certain probability. Essentially, LDA is a three-fold hierarchical Bayesian model. It can model the discrete data set and then finding the short descriptions to represent the statistical relations with the original data. Hence, the dimensions of the data set can be decreased by mapping the original data into the topic space. Further, it can be utilized by other machine learning approaches, such as classification, regression and clustering etc., for feature selection and parameter estimation. Figure 1 shows the graphical representation of the LDA model.

While using LDA on text data, a latent topic set is contained on the corpus. It means that each document can be represented as a mixture topic set. The processing of the whole text corpus is transferred to the processing of the topic sets. Hence, it reduces the dimension of corpus by text space mapping.

Given corpus 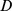 and document 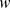, the generative process of LDA lists as follows:

1. Choose 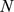 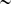
Poisson(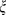). 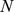 represents the length of a document.
2. Choose 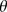 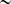 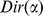. 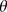 represents the occurrence probability of a topic.
3. For each of 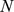 words 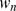:
  - Choose a topic 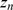 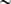 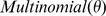.
  - Choose a word 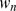 from 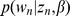, a multinomial probability conditioned on the topic 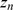.

The following shows the joint distribution of 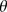, 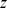 and 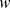 for the given 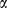 and 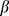:
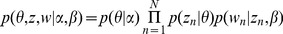
(1)The edge probability of Equation 1 at each document lists as follows:
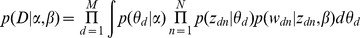
(2)

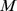
 is the number of documents in corpus 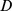. The expectation-maximization (EM) algorithm has been used in the parameter estimation step. For the length limitation of the paper, we will not present the details of the LDA model.

### Topic Clustering Approach

#### Topic Modeling

**Figure 2:**
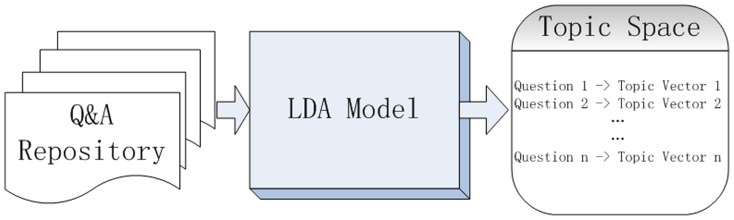
The transformation from documents to the representation of topic vector by the LDA model.

LDA is a generative probability model. In text processing, the LDA model can randomly generate topic sequences. Hence, the representation of each document can be transferred to a topic sequence. Figure 2 briefly presents the mapping progress from document space to topic space.

Here, in Figure 2, the document space indicates the Q&A repository in Yahoo! Answers. And the topic space can be represented as the corresponding topic vectors of the questions.

Based on the above descriptions, we then make the following assumptions:

*Assumption A*.

Each question 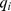 can be represented as a topic vector, in which its elements and their values indicate the topic distributions and the importance of the corresponding topics respectively.

*Assumption B*.

There exist a global function 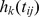, which indicates the “confidence” that the topic 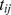 represents the semantic meaning of the question 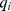, where 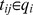.

We use 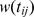 to represent the weight of the topic 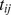 in the question 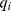. The larger the value of 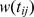, the more confidential of the topic 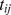 represents the semantic meaning of the question 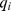. Instead of directly estimating 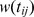, we employ a normalized variant of 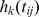 to derive the estimation:
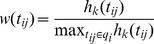
(3)To deduce the global function 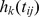, we utilize an unsupervised machine learning approach, which can seamlessly adopt the lexical information, topic distribution information and the topic weights information.

Next, we will introduce the features used for mining the similar questions from Yahoo! Answers Q&A repository. Table 2 gives the summary of the feature set used for measuring question similarity. We then detail these features as follows:

**Table 2:** A summary of the features used in finding similar question task.

Feature Name	Feature Description
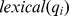	the lexical feature of the question 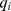 .
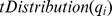	the topic distribution of the question 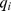 .
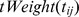	the weight of the topic 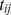 in the question 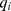 .

#### Features


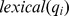
: We use the lexical information as the features for finding similar questions. It means that we capture the tokens in the question 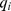 as one of the similarity metrics. Hence, we essentially adopt the advantages of the BoW approaches.


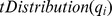
: As the topic model can transfer the Q&A pairs into the form of the topic vectors, we explore the distribution of the topics in question 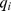. We thus capture the topic modeling information for question representation, further for the question similarity computing on topic space.


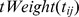
: As described in Equation (3), we also consider the weight of the topic 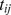 in the question 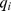. This is because the topics can represent the semantic meaning of the questions, and thus the topics assigned to the question 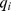 should not have the same importance. We utilize this feature to explore the difference among the topics in a given question.

#### Similarity Filter

Besides the question topic related information, we also consider the question content based factors to finding similar questions. It means that in our proposed approach, we also capture the string matching features. Moreover, we explore the linguistic analysis technique to mining the semantic similarity between the questions.

In this section, we plan to enhance the topic modeling based approach to finding similar questions by employing three filtering factors. They are question Levenshtein distance factor, part-of-speech (POS) sequence factor and word overlapping factor. We then utilize a unique function to combine the three factors for question similarity computing as follows:
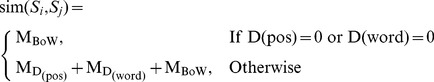
(4)Here, 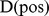 represents the Levenshtein distance between the POS sequences of two questions 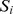 and 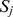. The definition of the Levenshtein distance can be summarized as:

*The minimum number of edit operations necessary to transform one string into another*.

The three edit operations are *Insert*, *Delete* and *Replace*.

Where, 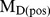 which is calculated as follows, indicates the similarity metric of the POS sequence factor.
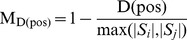
(5)

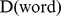
 represents the Levenshtein distance between question 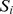 and 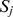. Here, we use the WordNet [26] for the automatic synonym identification which can be seen as the lexical level semantic expansion. Meanwhile, 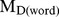 indicates the metric of the question Levenshtein distance, which can be calculated as:
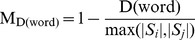
(6)

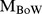
 represents the metric of the word overlapping factor. It can be obtained by computing the cosine similarity between 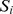 and 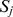. Meanwhile, we use the WordNet to expand the similar words in the questions for fuzzy matching. The 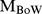 can be deduced as follows:
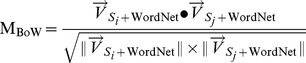
(7)


Finally, we empirically set the 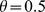 as the similar question filtering threshold. For a given question cluster, the questions 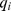 and 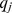 can be distinguished as similar questions, when the similarity between them is larger than 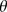.

#### A Unified Model for Finding Similar Questions

According to the above descriptions, we utilize the LDA model to gather the similar questions in topic space. We then propose a similarity filtering approach to enhancing the result of similar question exploring. In this section, we will give an overall conclusion about our proposed topic clustering based approach to finding similar questions. We summarize the following steps:

1. Preprocessing: Removing stop words and stemming.
2. LDA modeling: Transferring the Q&A repository into the corresponding topic vectors.
3. Topic guided clustering: Based on the three factors in Table 2, we utilize an unsupervised machine learning approach to clustering the questions into several clusters.
4. Similar question filtering: Selecting and reranking the similar questions for each of the clusters by using Equation (4).

**Figure 3:**
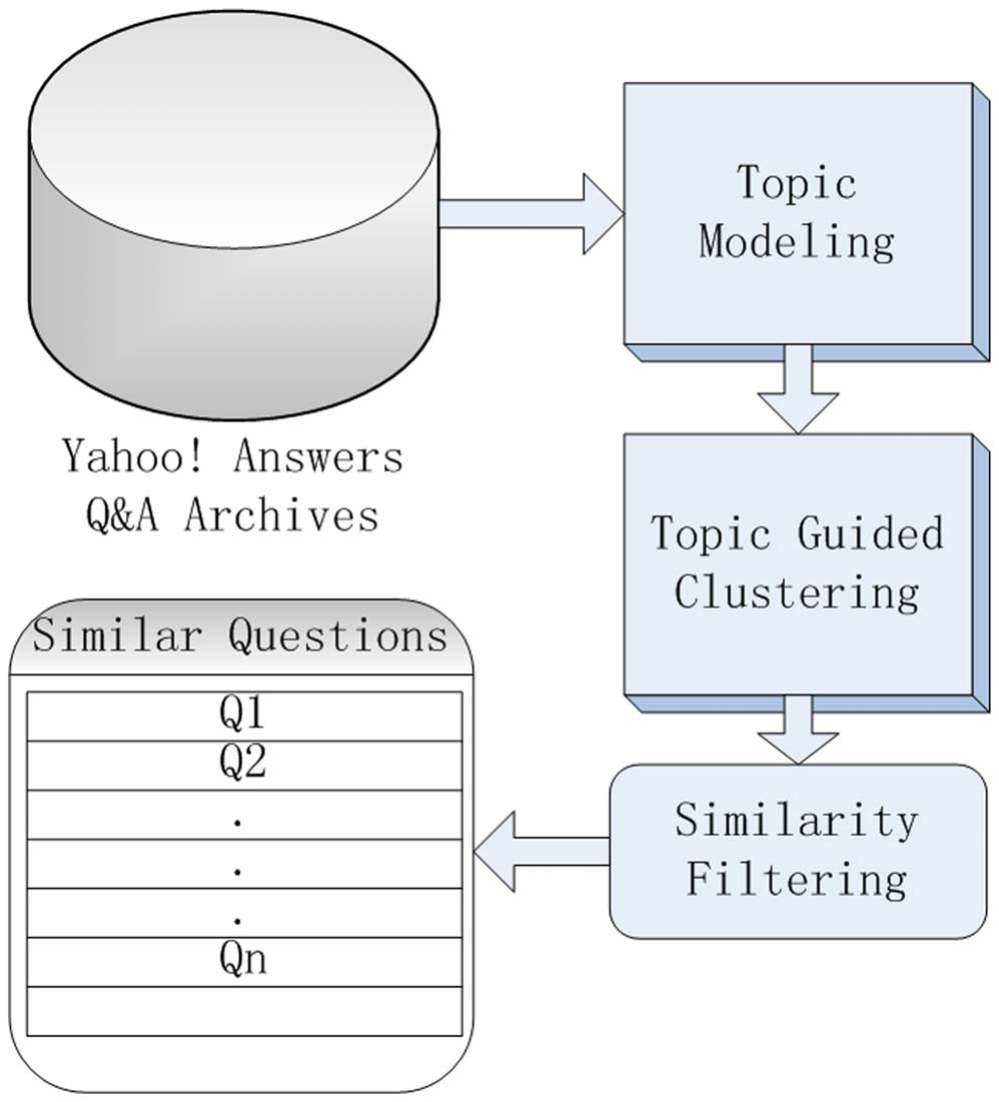
The framework of the proposed approach to finding similar question in Yahoo! Answers Q&A repository.

Figure 3 shows the framework of our proposed approach.

## Results and Discussion

### Data Set

We collected a total number of 1,123,034 questions from Yahoo! Answers using the Yahoo! Answers API (http://developer.yahoo.com/answers/). It covers a wide range of topics, including buying and selling, internet, etc. For each question, we extracted the question title, question content and chosen answers as the experimental data from the returned content by the API. From this data collection, we randomly select 10,000 questions as the queries and 200 as development set to tune the involved parameters. As the questions which are extremely short may represent ambiguous intent, we filtered out the questions which contain less than three terms through the random selection process. The experimental data is available at http://pan.baidu.com/share/link?shareid=343582&uk=2903372971.

To obtain the ground truth, we pooled top 20 relevance questions by utilizing various approaches, including vector space model, okapi BM25 model, language model, translation model, translation based language model [27], syntactic tree matching model [13], etc. We then asked two annotators, who were not involved in the design of the proposed method, to independently annotate whether the candidate question is similar (score 1) with the query question or not (score 0). When conflicts occurred, a third annotator was involved to make the final decision. We obtained a total number of 20,800 similar questions as the ground truth. Table 3 shows the statistics of our experimental data set.

**Table 3:** Statistics of the experimental data set.

# of queries	total # of questions	# of similar questions
10,000	1,123,134	20,800

### Evaluation

For the evaluation of our proposed approach, we introduce four experimental systems as the baselines respectively. The details of the comparing systems are as follows:

- BoW+Cluster (BC): BoW based similar question clustering approach which only use the term frequency (TF) and inverse document frequency (IDF) as features (baseline 1).
- BoW+Cluster+Filter (BCF): BoW based similar question clustering with our proposed similar filtering approach (baseline 2).
- updatedBoW+Cluster (upBC): Updated BoW based similar question clustering approach which use our proposed features(See Table 2) (baseline 3).
- SyntacticTreeMatching (STM): The state-of-the-art approach to finding similar questions in CQA repository which is proposed by [13] (baseline 4).
- LDA+Cluster (LDAC): Our proposed LDA model based similar question clustering approach with our proposed features.
- LDA+Cluster+Filter(LDACF): Our proposed LDA based similar question clustering approach which is also integrating the proposed similar filtering approach.

For baseline 4, we run the original syntactic tree matching system to finding similar questions in our data set. We use average precision (AP) and precision at position one (p@1) to evaluate the performance of the comparing systems. Table 4 shows the experimental results of the above systems. In Table 4, all the models are evaluated by the average precision (AP) and precision at position one (p@1). Here, all the scores are the real values in the two evaluating measurements. Furthermore, we also capture the percentage of AP improvements. Here, the t-test works for testing the statistical significance on finding similar question result which contains a large number of questions. As the t-test works for the non-normal data only if the sample size is large, the t-test used in our experimental data set is rational.

**Table 4:** Experimental results of the comparing systems for finding similar questions.

Models	BC	BCF	upBC	STM	LDAC	LDACF
AP	0.543	0.556	0.564	0.575	0.638	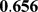 †
% AP improvements over						
**BC**	N/A	+2.39	+3.87	+5.89	+17.50	+20.81
**BCF**	N/A	N/A	+1.44	+3.42	+14.75	+17.99
**upBC**	N/A	N/A	N/A	+1.95	+13.12	+16.31
**STM**	N/A	N/A	N/A	N/A	+10.96	+14.09
**LDAC**	N/A	N/A	N/A	N/A	N/A	+2.82
p@1	0.550	0.561	0.577	0.585	0.648	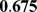 †

† indicates the results of our proposed methods are statistical significance over the four baseline methods (within 0.95 confidence interval using the 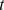-test). The results of our proposed approach are in bold.

From Table 4, we have the following observations:

First, to compare the performance between the **BC** and **BCF**, we can observe that the similar filtering approach is effective to finding similar questions. This is because, in our proposed similar filtering approach, we actually consider the strict tokens matching factors and the linguistic analysis information of the questions.

Second, to see the results of the **BC** and **upBC**, we can conclude that our proposed features outperform the statistical based features (TF and IDF) for similar question clustering. This is because that our proposed features not only take the lexical information into consideration, but also consider the topic distribution and weight for the questions. Hence, the similarity modeled by our proposed features is enhanced by combining the topic space similarity between the questions.

Third, we can observe that our proposed **LDACF** approach outperforms the BoW based methods. It demonstrates that, the topic modeling approach can better represent the Q&A data than the BoW methods and further better captures the similarity between the questions. This is because that our proposed approach captures the similarity of the questions not only through the lexical and linguistic information, but also mapping the Q&A text into the topic space. We thus measure the semantic similarity of the questions in the topic level.

Fourth, to comparing with the **STM** and **LDACF**, we can see that our proposed **LDACF** outperforms the **STM**, which is the state-of-the-art approach in finding similar questions in the CQA archives. This is because the **STM** approach only considers the syntactic tree structures. It employs the tree kernel function to measure the question similarity. However, it neither introduces the fuzzy matching scheme, nor considers the semantic similarity in topic level. In our proposed approach, we capture the questions' semantic similarity in the topic space. And we also take the advantages of the lexical and linguistic analysis techniques.

Furthermore, to analyzing the experimental results, we found that there exist the clusters that contain only one question. We remove these clusters in the similar question clustering result and evaluate the proposed approach again. Table 5 shows the upper bound of the evaluation data which is obtained by removing the error clusters in similar question clustering results.

**Table 5:** The upper bound of the evaluation data which is obtained by removing the error clusters in similar question clustering results.

	BC	LDACF
Upper bound of evaluation data	93.7%	99.1%

From Table 5, we can see that the number of generated error clusters by the **LDACF** is less than **BC**. It demonstrates that the topic guided clustering approach is better than the BoW based clustering approach in error cases handling. It is because those topics can be seen as a higher level lexical semantics. Hence, the clustering results of **LDACF** is more accuracy and robust than the **BC**.

We also compare the performance between the **BC** and **LDACF** in the refined data set. For evaluation, we also employ the average precision (AP) and precision at position one (p@1). Table 6 shows the experimental results of the above two approaches in the refined data set.

**Table 6:** The experimental results of the **BC** and **LDACF** approaches in the refined data set.

	BC	LDACF
AP	0.58	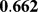 *
p@1	0.587	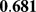 *

* indicate that the results of the **LDACF** are statistical significance over the **BC** (within 0.95 confidence interval using 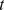-test).The results of our proposed approach are in bold.

### Evaluation On Diverse Data sets

To check the effectiveness of our proposed approach, we also test the performance on another two data sets. The first data set is used to evaluate the performance of question retrieval in [28]. We employed the labeled question and answer pairs to test the performance of our proposed approach. There is a total number of 252 question and answer pairs in the annotated data set, which can be obtained at http://homepages.inf.ed.ac.uk/gcong/qa/annotation_result.txt. The second data set is collected from Twitter (https://twitter.com/) by using the Twitter API (https://dev.twitter.com/docs/using-search). We collected a total number of 13,683,354 questions from Twitter as our experimental data set. (The Twitter API has the command to allow people obtain questions from Twitter). It covers a wide range of topics, including famous people, internet, makeup etc. From the data set, we randomly select 200 questions as queries and 100 questions as development set to tune the involved parameters. For preprocessing, we filtered out the non-English characters and the urls.

To obtain the ground truth, we pooled top 20 relevance questions by utilizing various approaches, including vector space model, okapi BM25 model, language model, translation model, translation based language model [27], syntactic tree matching model [13], etc. We then asked two annotators, who were not involved in the design of the proposed method, to independently annotate whether the candidate question is similar (score 1) with the query question or not (score 0). When conflicts occurred, a third annotator was involved to make the final decision. We obtained a total number of 678 similar questions as the ground truth.

For the evaluation, we utilize the average precision (AP) and precision at position one (p@1) on the above two data sets. Table 7 shows the experimental results on the diverse data sets.

**Table 7:** Experimental results of comparing systems on the diverse data sets for finding similar questions.

Models	Cong et al.		Twitter	
	AP	p@1	AP	p@1
**BC**	0.517	0.520	0.551	0.570
**BCF**	0.525	0.532	0.559	0.570
**upBC**	0.533	0.544	0.577	0.585
**STM**	0.554	0.560	0.593	0.600
**LDAC**	0.598	0.615	0.608	0.620
**LDACF**	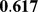 †	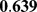 ‡	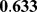 †	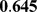 ‡

† and ‡ indicates the results of our proposed methods are statistical significance over the four baseline methods (within 0.95 confidence interval using the 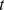-test). The results of our proposed approach are in bold.

From Table 7, we can see that our proposed approach outperforms the four baselines on the above two data sets. It demonstrates that our proposed approach can adapt to the diverse data sets and perform well on finding similar question task. To compare the experimental results on the data sets of Cong et al. and Twitter, we can see that the results on Twitter data outperform those on Cong et al. data. This may be because that the questions on Twitter are extremely short. After the preprocessing, the average length of the Twitter questions by terms equals to 3.2. While the questions in Cong et al. data contain more terms than Twitter questions. Meanwhile, we also observe the experimental data on Twitter questions. We found that, after preprocessing, the reserved terms of Twitter questions are usually named entities, such as Iphone, Xbox, Barack Obama, Android, Chanel, Clinique etc. Hence, the users' intent can be clearly represented on Twitter questions so that the precisions of finding similar questions are higher than that on Cong et al. data.

### Topic Number Analysis

**Figure 4:**
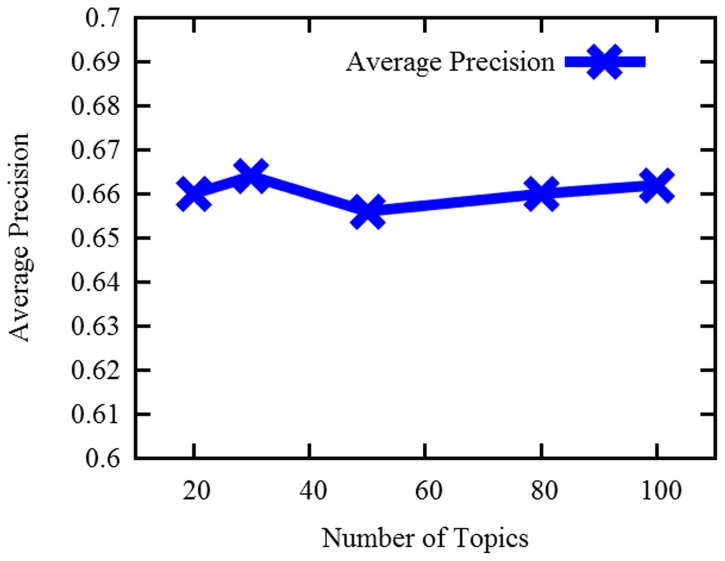
The change of the average precision with the varying of the topic numbers.

In this section, we plan to verify the influence of the topic number on the final results of finding similar questions. We then run our proposed system in the various topic numbers. Figure 4 shows the change of the average precision when varying the number of the topics.

From Figure 4, we can see that the average precision varies a little when changing the number of the topics. Hence, it demonstrates that our proposed approach is not sensitive to the number of the topics.

### Error Analysis

In the paper, we proposed an unsupervised machine learning approach to finding similar questions in Yahoo! Answers Q&A repository. Although our experiment results outperform the baselines significantly, the final results also need to be further improved. Hence, we analyze the experiment results and we then conclude the following errors which influence the performance of finding similar questions.

- Although we employed LDA model to capture the topic information, there are a large amount of new entities in our Q&A repository. As we have no entity recognition module, our approach fails to deal with the specific name entities. For example, the similar questions “Where can I find a cheap canon 60D camera?” and “Where is the cheapest online source to buy canon 500d?”, our approach cannot distinguish the “canon 60D” and “canon 500d” as both of the questions' topic is “canon”.
- For the diversity representation of the user questions, there exist the questions with ambiguous meanings. However, the question content and the chosen answers can be used to clarify the question meaning. For example, “Blackberry?” and “How do i fix it?” are two questions which share no common words. Through the analysis of the question content, we found that both of the two questions are asking for the “camera light” problem. Hence, how to mine the question similarity between two questions that share none or fewer common words is a non-trivial task.

### Sample Analysis

In order to illustrate the topic distribution in our experiment data set, we list several topic words with high probability as follows. Table 8 shows the four topics which are mining from our experiment data by using the topic modeling approach. Furthermore, to verifying the effectiveness of the topic modeling approach, Table 8 shows the distribution of the topics in the example question.

**Table 8:** Four topics and the words mined from our experiment data set.

TOPIC 1 (*t*1)	TOPIC 2 (*t*2)	TOPIC 3 (*t*3)	TOPIC 4 (*t*4)
E71	Music	Card	internet
Nokia	player	memory	wifi
E63	firmware	phone	connect
work	update	PC	connection
phones	device	file	WLAN
cheaper	version	transfer	access
N97	media	computer	home
E51	latest	contacts	settings
5730	problem	folder	point
features	quality	suite	wireless
prefer	sound	bluetooth	laptop
LG	files	data	working
Phone	software	cable	password
Black	reason	copy	router
	information	format	Wi
	flash	installed	
	refresh	USB	
		Songs	

My [Nokia E71]*_t_*_1_ [music player]*_t_*_2_ is not [working]*_t_*_4_ properly even restored to factory [setting]*_t_*_4_. How can I fix this [problem]*_t_*_4_? After I install some added [features]*_t_*_1_ on my [Nokia E71]*_t_*_1_, it started not to [work]*_t_*_1_ properly. Having it restored to default factory [settings]*_t_*_4_, my [music player]*_t_*_2_ is not [working]*_t_*_4_ properly afterwards (was [working]*_t_*_4_ before restoring). Please help me resolve this issue, it would be highly appreciated Thanks!

From Table 8, we can see that the generated topics, which can be distinguished by the different colors, can well represent the meaning of the example question. Hence, it also demonstrates that our proposed topic guided clustering approach to finding similar questions is rational and effective.

### Limitations of the study, open questions, and future work

In this section, we will discuss the limitations of this study. We would like to thank the anonymous reviewer for the comment of which we should test our approach on a diverse range of data sets. To check the effectiveness of our proposed approach, we will test our proposed approach on more available data sets in future work. Meanwhile, we would like to thank the anonymous reviewer for the comment on feature fusing. In future work, we will consider to automatically fusing the features in finding similar questions. Inspired by [29], [30], we plan to learning the different feature weights by using several models.

We also noticed that the precision of our proposed approach is still low. In our future work, we plan to employ the name entity recognition scheme so that we can accurately identify topic related terms. We will utilize more semantic resources, such as phrase based paraphrasing and translation based synonym extracting, to capture the lexical semantic similarity. Specifically, we will try to reformulate the original query question into the semantic similar questions using the approach which is proposed by Zhang et al. [5]. To utilize these reformulations as the extended queries, we can obtain more similar questions and thus increase the recall rate of the similar question finding task.

## Conclusions

In this paper, we proposed a topic guided clustering approach to finding similar questions in CQA archives. We utilized the LDA model to map the Q&A data set into topic space and took the advantages of the topic modeling as guided information to cluster the questions which share the same or similar topics. We verified that the LDA guided clustering approach significantly outperformed the state-of-the-art approach of finding similar questions in CQA archives as well as other baselines.

Our proposed topic clustering approach can adapt to other applications. In future work, we plan to use our topic clustering for finding correlated salient object segments [31]–[33] from large image collections as it directly enables a number of interesting image retrieval and manipulation and landmark identification applications [34]–[37].
